# Test Preparation in Figural Matrices Tests: Focus on the Difficult Rules

**DOI:** 10.3389/fpsyg.2021.619440

**Published:** 2021-04-15

**Authors:** Kai Krautter, Jessica Lehmann, Eva Kleinort, Marco Koch, Frank M. Spinath, Nicolas Becker

**Affiliations:** Personality Psychology and Psychological Assessment, Saarland University, Saarbrücken, Germany

**Keywords:** figural matrices test, test preparation, construction rules, training study, intelligence assessment

## Abstract

It is well documented that training the rules employed in figural matrices tests enhances test performance. Previous studies only compare experimental conditions in which all or no rules were trained and therefore ignore the particular influence of knowledge about the easy and difficult rules. With the current study, we wanted to provide some first insights into this topic. Respondents were assigned to four groups that received training for no rules, only the easy rules, only the difficult rules, or for all rules. The results show that a training only for the difficult rules was more effective than the other trainings. This applies also to performance in the easy rules that were actually not part of the training. A possible explanation for this finding is a facilitation of the solution process that is primarily driven by knowledge about the difficult rules. In conclusion, our results demonstrate that taking differences between the rules into account may provide a deeper understanding of the effects of trainings for figural matrices tests.

## Introduction

This study addresses the way test preparation influences the performance in figural matrices tests. To this extent, the particular influence of knowledge concerning the easy and difficult rules is examined.

Figural matrices are a very common item format used to assess reasoning and are regarded as one of the best indicators of general intelligence (Marshalek et al., 1983; Carpenter et al., 1990; Jensen, 1998; but see also Gignac, 2015). Intelligence is related to a plethora of important variables in everyday life (Brand, 1987; Neisser et al., 1996; Gottfredson, 1997, 2004; Jensen, 1998). Intelligence measures are particularly useful to predict educational (Roth et al., 2015) and vocational success (Schmidt and Hunter, 1998) and are therefore often part of high-stakes tests for student and personnel selection. Because the test results have high importance for the life of the respondents, test preparation is an issue that needs to be taken into account (Buchmann et al., 2010). Previous studies indicate that test preparation can lead to substantial score gains (Kulik et al., 1984; Hausknecht et al., 2007; Scharfen et al., 2018) that do not reflect changes in ability (te Nijenhuis et al., 2007; Estrada et al., 2015) what might negatively influence test validity. A further problem associated with test fairness is the fact that test preparation materials are often expensive and thus not available for financially underprivileged respondents. A deeper understanding of the influence of test preparation on the test performance of respondents is therefore warranted. In the context of figural matrices tests, test preparation consists of teaching the respondents the rules that are commonly used to construct the items (Loesche et al., 2015; Schneider et al., 2020).

**FIGURE 1:**
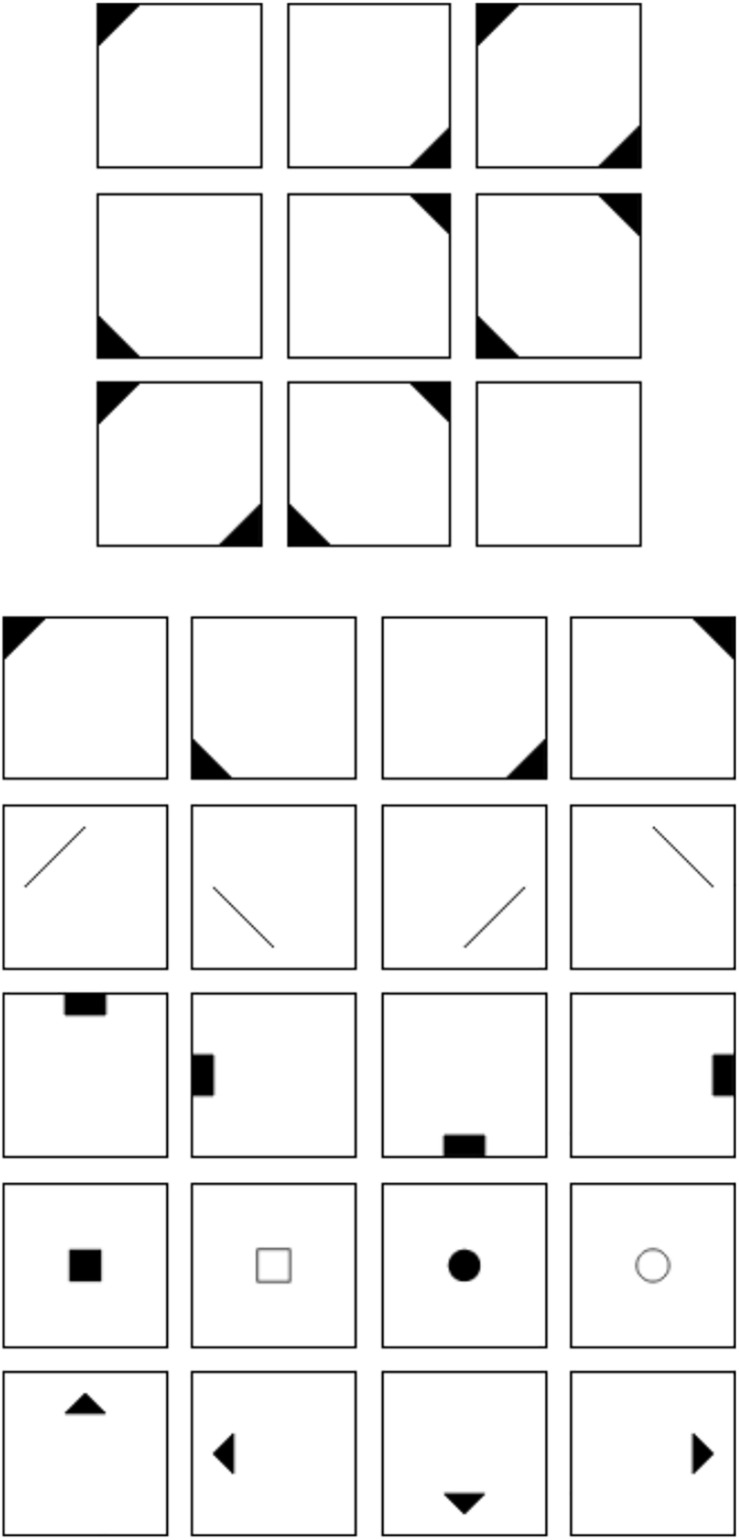
Figural matrix item.

Figure 1 shows an example of a matrix item used in the current study. The item stem can be found in the upper half of Figure 1. It consists of a 3 × 3 matrix filled with geometrical symbols. The elements follow specific rules across the rows of the matrix. In the case of the example in Figure 1, the elements of the first and second cell of a row sum up in the third cell of the row. The last cell at the bottom right cell of the matrix is left empty. The task of the respondents is to fill this cell with the symbols that logically complete the matrix. We used a distractor-free response format (cf. Becker et al., 2015) that can be found in the lower half of Figure 1. It consists of 20 symbols from which the respondents have to choose those individual symbols, which together form the correct solution. In the case of the example in Figure 1, the correct solution would be the four symbols in the first row of the response format. We decided to choose a distractor-free format because we wanted to analyze the results on the level of single rules. When using a distractor-based response format, it is usually only possible to determine whether the item as a whole was solved correctly. Furthermore, it has been demonstrated that the construct validity of figural matrices tests is higher when a distractor-free response format is used (most presumably due to the prevention of response elimination strategies; cf. Arendasy and Sommer, 2013; Becker et al., 2016). We would therefore argue that the generalizability is higher when distractor-free response formats are used.

**FIGURE 2:**
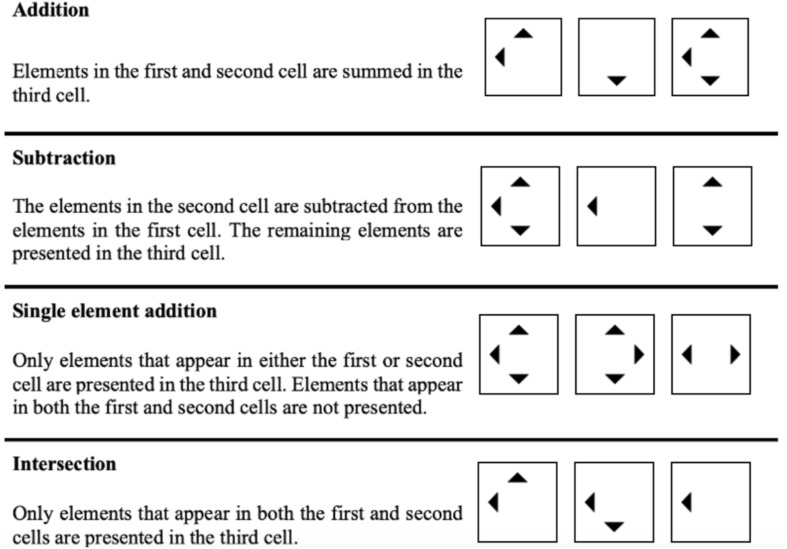
Rules employed in the current study.

Figure 2 illustrates the four rules employed in the current study. They were chosen because they are commonly used and because several studies show that the difficulty of matrix items is determined by these rules (e.g., Embretson, 1995; Hornke et al., 2000; Arendasy and Sommer, 2005; Freund et al., 2008; Becker et al., 2016). Furthermore, addition and subtraction are regarded as easier than single element addition and intersection (cf. Vodegel Matzen et al., 1994; Embretson, 1998; Preckel and Thiemann, 2003; Arendasy et al., 2016; Krieger et al., 2019).

Prior research has demonstrated that test preparation can increase the respondents’ test scores (for an overview, cf. Arendasy et al., 2016). In connection with figural matrices, Loesche et al. (2015) and Schneider et al. (2020) demonstrated that respondents perform better when being taught all rules used in the test. A shortcoming of these studies is that there was no experimental condition in which only some of the rules were taught. Therefore, it was not possible to analyze the influence of test preparation for the single rules.

The purpose of this study is to identify the particular contribution that teaching the easy (addition, subtraction) and difficult rules (single element addition, intersection) has on the improvement in the test. Furthermore, we wanted to study possible transfer effects between knowledge concerning the rules that were not learned. We did this by assigning the respondents to four groups that received training for none of the rules (no training group), only addition and subtraction (easy rules group), only intersection and single-element addition (difficult rules group), or all four rules (full training group). Following the results of the previous studies, we expected that teaching the rules generally increases test performance. At a more differentiated level, we expected that teaching the easy rules is less effective than teaching the difficult rules, while a training for all rules should be most effective (H1. no training group < easy rules group < difficult rules group < full training group). With respect to transfer effects between the rules, we expected that, apart from explicit knowledge concerning the specific rules, test preparation might also result in a deeper understanding of the general principles of the test (e.g., that there are rules, that rules affect certain symbols). Therefore, respondents in the easy rules group should – compared to the no training group – show a better performance in the difficult rules although they were not taught to them (H2. Performance on difficult rules: easy rules group > no training group). Likewise, respondents in the difficult rules group should show better performance in the easy rules than respondents in the no training group (H3. Performance on easy rules: difficult rules group > no training group).

## Materials and Methods

### Procedure and Participants

Participants were approached by sending them links *via* e-mail or social media (e.g., Whatsapp, Instagram). In return for their participation, participants could receive an individual feedback concerning their performance in the test and/or win one of 49 gift vouchers of 10 EUR in a lottery. The link led the participants to a website on which the test took place. The participants initially had to provide informed consent and to complete a demographic questionnaire. Next, they received a general instruction on how to solve a matrices test. To become more familiar with the item stem and the distractor-free response format, they were provided with a simple example item following a rotation rule that was not used in the actual test. After the general instruction, participants were randomly assigned to the four treatment conditions. Participants in the no training group started the test without any further information. Before starting the test, participants in the easy rules group received information on the rules addition and subtraction, participants in the difficult rules group on intersection and single element addition and participants in the full training group on all four rules (cf. the translated version of the training in the Electronic supplementary material; ESM).

A total amount of 299 respondents started the test. However, 12 respondents (4%) had to be excluded since they did not complete all of the items. The final sample consisted of *n* = 287 respondents (63.2% women, 0.7% diverse, 36.1% men). The mean age was *M* = 26.30 years (SD = 10.20; 18≤ age ≤62). The vast majority (88%) had A-levels (German Abitur) or higher educational qualifications. Seventy-four (25.78%) participants were assigned to the no training group, 68 (23.69%) to the easy rules group, 78 (27.18%) to the difficult rules group, and 67 (23.33%) to the full training group.

### Figural Matrices Test

Because we wanted to analyze the test results at the level of single rules, we used figural matrices with a distractor-free response format (cf. Becker et al., 2015). A rule was regarded as correctly solved when the relevant symbols were chosen from the response format.

Following the common practice, the matrices test was administered as a power test. To ensure time economy and to ensure that every respondent had the opportunity to work on every item we nevertheless implemented a time limit of 90 s per item. This time limit was determined based on response times in an earlier study (Becker et al., 2015). In this study, respondents responded well below 90 s even when no time limit was given.

Overall, we constructed 26 items. The rules employed in the items can be found in Supplementary Table 1 of the Supplementary Material. Every possible permutation of the four rules was realized at least once. To ensure comparability, each of the rules was used 19 times, which results in a total of 76 rules throughout the test. The internal consistency was considerably high in the whole sample (α = 0.98) as well as in the four subgroups (0.97≤α ≤0.99).

### Statistical Procedure

All statistical analyses were carried out using R 4.0.3 (R Core Team, 2020). To ensure that differences between the performance in the different training groups were not due to differences in the factor structure, we first estimated measurement invariance by computing a series of multigroup confirmatory factor analyses (MGCFAs). To conduct the multiple-group analyses, the items were summed into three parcels with comparable mean factor loadings that were based on the results of an exploratory factor analysis. Following the guidelines of Hirschfeld and von Brachel (2014), we computed four models in which equality constraints were applied to the number of latent variables and their loadings on the indicators (configural model), the magnitude of the factor loadings (weak invariance model), the factor loadings and intercepts (strong invariance model), and the factor loadings, intercepts, as well as the residuals (strict invariance model). Strong invariance (indicated by insignificant χ^2^ difference tests between the first three models) is particularly important when latent correlations are being compared between groups (Chen, 2008).

Before testing the hypotheses, we inspected the median (*Mdn*), interquartile range (IQR), skewness, and kurtosis of the distribution of test performance in the different training and rule groups as well as in the overall group. Furthermore, we used the Shapiro–Wilk test to assess the normality of the data and the Fligner-Killeen test to assess the homogeneity of variance between the treatment conditions. Because the distribution parameters partially indicated a deviation from normality and as both the Shapiro–Wilk as well as the Fligner-Killeen test were significant (see section “Results”), we relied on non-parametric statistics and computed a rank-based analysis of variance-type statistics (ATS; Erceg-Hurn and Mirosevich, 2008) using the nparLD package (Noguchi et al., 2012). We conducted a 4 × 2 ATS (between-subjects training factor: no training group vs. easy rules group vs. difficult rules group vs. full training group) (within-subject rules factor: easy rules vs. difficult rules). Following the guidelines of Brunner et al. (2002), the denominator degrees of freedom were set to infinity because using finite denominator degrees of freedom might lead to a higher type I error (cf. Bathke et al., 2009). The dependent variable was the percentage of rules correctly solved in the test. H1 was evaluated by computing a *F*-test for the between-subjects factor and by comparing the treatment conditions in pairwise *post hoc* tests. To quantify the size of this effect, we computed the rank-based effect size measure Cliff’s *d* (Cliff, 1993) using the effsize package (Torchiano, 2020). To test H2 and H3, we computed the relative treatment effects (RTE) (cf. Brunner et al., 2019) for the difficult rules in the easy rules group and the no training group and for the easy rules in the difficult rules group and the no training group. The RTE can be calculated by the quotient of the mean rank of each group and the number of ranks in total, which was 574 (two data points for each of our 287 participants). Following Field and Iles (2016) and using the confidence intervals around the RTEs, we regarded an overlap smaller than half of the length of the average margin of error (MOE) as an indicator of a significant difference between the RTEs. In addition, we computed a Cohen-like effect size (*d*_*RTE*_) by subtracting the two RTEs from each other and dividing them by the pooled standard deviation.

## Results

The outputs of the MGCFA models, including all factor loadings and model comparisons, are reported in the ESM. None of the χ^2^ difference tests between the different MGCFA models was significant [configural vs. weak: Δχ^2^(6) = 4.38, *p* = 0.62; weak vs. strong: Δχ^2^(6) = 8.40, *p* = 0.21; strong vs. strict: Δχ^2^(9) = 14.76, *p* = 0.10]. Given this fact, differences between the performance in the different training groups cannot be attributed to different factor structures of the test.

The distribution parameters of test performance in the different training and rule groups as well as in the overall group partially indicate a deviation from normality (see Table 1). The Shapiro–Wilk test showed that the data was not normally distributed per group [*W*(287) = 0.72; *p* < 0.01]. A significant Fligner-Killeen test [χ^2^(5, 287) = 63.95; *p* < 0.01] indicated different variances in the subgroups.

**TABLE 1:** Medians and interquartile ranges (IQRs) of correctly solved rules depending on each rule and training group.

	Easy rules	Difficult rules	Overall	Skewness	Kurtosis
Difficult training	0.95 [0.89; 0.97]	0.95 [0.89; 0,97]	0.95 [0.89; 0.97]	–3.1	9.27
Full training	0.95 [0.89; 0.97]	0.92 [0.79; 0.95]	0.92 [0.80; 0.96]	–1.9	2.48
Easy training	0.92 [0.76; 0.97]	0.87 [0.62; 0.92]	0.88 [0.69; 0.94]	–1.41	0.86
No training	0.89 [0.69; 0.95]	0.84 [0.40; 0.95]	0.88 [0.57; 0.95]	–0.96	–0.45
Overall	0.92 [0.76; 0.97]	0.87 [0.55; 0.95]	0.92 [0.76; 0.96]	–1.65	1.56

*Medians can be found in front of the brackets, bounds of the IQRs in the brackets. The last two columns show the skewness and kurtosis of the data distribution per group.*

Regarding H1, the main effect of the between-subjects factor was significant [F_*ATS*_(2.97, ∞) = 7.11; *p* < 0.01]. Table 1 shows the median values of the percentage of rules solved in each group as well as the corresponding IQRs. It can be recognized that the difficult rules group (*Mdn* = 0.95) performed best followed by the full training group (*Mdn* = 0.92), while the easy rules group (*Mdn* = 0.88) and the no training group (*Mdn* = 0.88) solved less rules. Pairwise *post hoc* tests revealed that the full training group showed significant differences to the difficult training group [t_*A*__*TS*_(1) = 5.28, *p* = 0.02, Cliff’s *d* = −0.23], but no significant differences to the no training group [t_*ATS*_(1) = 3.50, *p* = 0.06, Cliff’s *d* = −0.19], and the easy training group [t_*ATS*_(1) = 2.52, *p* = 0.11, Cliff’s *d* = −0.17]. The difficult training group differed significantly from the easy training group [t_*ATS*_(1) = 15.38, *p* < 0.01, Cliff’s *d* = −0.38] and the no training group [t_*ATS*_(1) = 17.72, *p* < 0.01, Cliff’s *d* = −0.39]. The difference between the easy training group and the no training group was not significant [t_*ATS*_(1) = 0.08, *p* = 0.77, Cliff’s *d* = −0.04].

**FIGURE 3:**
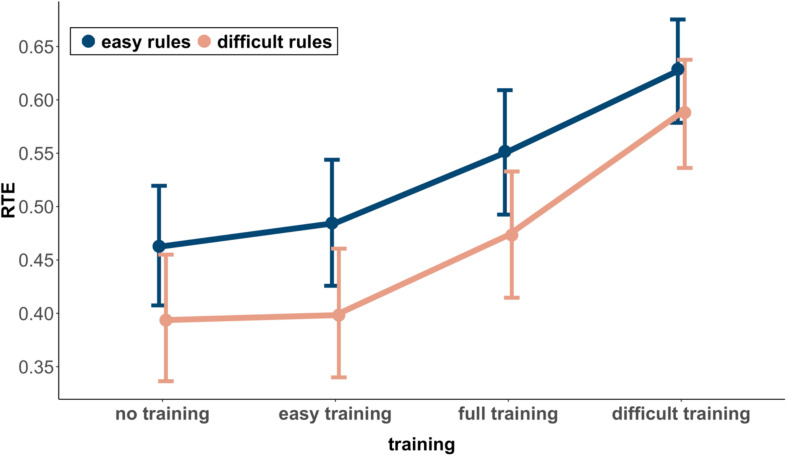
Relative treatment effect (RTE) and 95% CI among training groups and rules.

Figure 3 shows the RTEs and corresponding 95% confidence intervals for the easy and difficult rules in the four groups. Concerning H2, it can be seen that the RTE of the difficult rules in the easy rules training group does not differ substantially from the RTE of the difficult rules in the no training group (RTE = 0.40 vs. RTE = 0.39). Because the overlap (0.12) was larger than half of the length of the average MOE (0.5 × MOE = 0.06, *d*_*RTE*_ = 0.02), the difference was not significant. With respect to H3, the RTE of the easy rules in the difficult rules training group was substantially larger than the RTE of the easy rules in the no training group (RTE = 0.63 vs. RTE = 0.46). With an overlap (<0.01) smaller than half of the length of the average MOE (0.5 × MOE = 0.05, *d*_*RTE*_ = 0.79), this difference was significant.

## Discussion

Our goal was to study the influence of a brief rule training on the performance in a figural matrices test. In addition to prior studies, which only considered the difference between groups that received no training or a training for all rules, we wanted to identify the particular contribution of a training for either the easy or the difficult rules and possible transfer effects between them.

With respect to H1, the results show that the type of training significantly influenced test performance. Contrary to our initial assumptions, however, the difficult rules training group performed better than all other groups. It is especially remarkable that they even outperformed the full training group, which received a more extensive training that focused on the difficult and the easy rules. The fact that the easy rules training group did not perform better than the no training group leads us to suggest that knowledge concerning the easy rules only has a minor influence on the solution process. An explanation for the finding that the difficult rules training group performed better than the full training group might therefore be that respondents receiving information on all four rules pay less attention to the difficult rules and therefore profit less than respondents who receive only information on the difficult rules.

Concerning H2, we did not find a transfer effect from the easy to the difficult rules. Therefore, it is unlikely that respondents profit from knowledge about the general principles of the test. Interestingly, the evaluation of H3 showed that the respondents in the difficult rules training group solved significantly more easy rules than the no training group. Because H2 was not confirmed, interpreting this finding as a transfer effect is not plausible. Instead, these results again suggest that knowledge about difficult rules has a stronger influence on the solution process than knowledge about easy rules. A training for the difficult rules would facilitate the whole solution process more strongly than a training for the easy rules. In turn, this would lead to a better performance of the difficult rules training group in both the difficult as well as the easy rules.

A limitation of the current study is that the sample had a rather high and homogeneous ability. We did not expect this since previous studies conducted with comparable tests in comparable samples (e.g., Becker et al., 2015, 2016) showed a lower mean test performance and a higher variance. Albeit this limitation, we would argue that there was still sufficient variability between the respondents as indicated by the width of the IQRs of test performance. Given the fact that we used non-parametric tests, which correct for the skewness of the data, and that we found substantial effect sizes, we would conclude that our findings are rather robust. Nevertheless, the influence of the easy rules on the solution process might have been attenuated by the fact that all respondents already had rather good prerequisites to solve the easy rules. With this in mind, it would be premature to conclude that training the easy rules generally has no influence on the performance in the test. Instead, it would as always be important to conceptually replicate this study with an improved design. Such a study should use a sample that is more diverse with respect to the respondents’ abilities. Low-ability respondents might indeed profit from a training for the easy rules, while we would expect that the results for the high-ability respondents show the same pattern as in the current study.

A reviewer brought up the interesting question if there might be a differential impact of training on items using different combinations of easy and difficult rules. A recent study (Krieger et al., 2019) from our research group dealt with a similar issue. We found out that performance differences between items with single and multiple rules are mainly driven by different filtering demands (i.e., the ability to focus on symbols related to a certain rule and to ignore the other ones). We would expect that our training does not influence filtering ability. Therefore, our hypothesis would be that there is no differential impact of training on performance differences between items with only easy rules and items with easy and difficult rules. It would of course be necessary to substantiate this assumption in an empirical study. Unfortunately, our study included only six items with only two rules, which additionally showed strong ceiling effects. Therefore, the current dataset is not suitable to dig deeper into this question. Nevertheless, a study especially designed to evaluate possible differences of the impact of training on items with different combinations of easy and difficult rules would be interesting endeavor for future research.

Taken together, our results show that the effects of training the rules used in figural matrices tests are more differentiated than the results of previous studies suggest. Although we would conclude that training the rules positively affects performance in figural matrices tests, we would nevertheless also argue that future studies should analyze which respondents (high vs. low ability) benefit from which type of training (difficult vs. easy rules). Corresponding findings could be used to develop tailored trainings that enable different types of respondents to show their full potential in the test. This in turn would possibly level out differences associated with test preparation in order to tackle the negative influences of test preparation on test validity and test fairness that were mentioned in the “Introduction.”

## Data Availability Statement

The datasets presented in this study can be found in online repositories. The names of the repository/repositories and accession number(s) can be found below: https://osf.io/nc3us/.

## Ethics Statement

Ethical review and approval was not required for the study on human participants in accordance with the local legislation and institutional requirements. The patients/participants provided their written informed consent to participate in this study.

## Author Contributions

KK, JL, EK, and NB: conceptualized the study, gathered the sample, conducted the statistical analyses, and wrote the manuscript. MK: developed the test environment and edited and approved the manuscript. FS: edited and approved the manuscript. All authors contributed to the article and approved the submitted version.

## Conflict of Interest

The authors declare that the research was conducted in the absence of any commercial or financial relationships that could be construed as a potential conflict of interest.
