# Supplementary material for: Test Preparation in Figural Matrices Tests: Focus on the Difficult Rules
Source: Front Psychol. 2021 Apr 15;12:619440. doi: 10.3389/fpsyg.2021.619440 (PMC8081851; doi:10.3389/fpsyg.2021.619440)
Supplement: Supplementary file 1 [file Data_Sheet_1.pdf]

Rule training (translated from german)

Rules

In a matrices test, one or more rules are applied. Please read through the following potentially occurring rules and apply them during the test. Note that they may appear individually or in combination in a task.

Addition

The elements of the first and second cell are combined. Therefore, the third cell contains both elements.

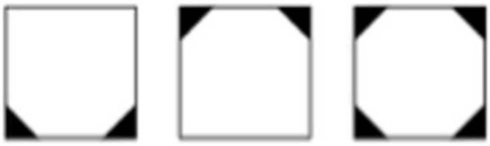

The first cell contains the two lower corners, the second cell contains the two upper corners. The third cell thus contains all four corners.

Subtraction

The elements of the second cell are removed from the elements of the first cell, so that only the remaining elements are displayed in the third cell.

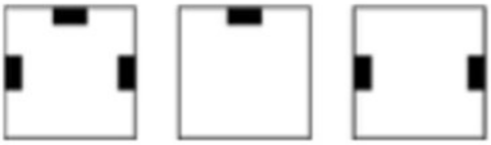

In the first cell, the left, top and right boxes are shown. In the second cell, the box at the top is shown. If the content of the second cell (upper box) is now removed from the elements of the first cell, the third cell is obtained. This cell contains two boxes: left and right.

Single Element Addition

Elements present in both the first and second cells cancel each other out. Thus, the third cell contains elements that are mapped either only in the first cell or only in the second cell.

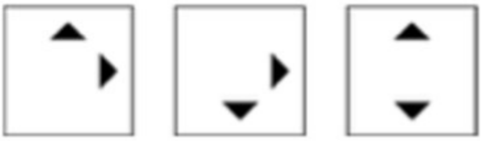

The triangle at the top is mapped only in the first cell, the triangle at the bottom only in the second. These triangles are thus mapped in the third cell. The triangle at the right edge appears in both the first and the second cell and is thus no longer mapped in the third cell.

Intersection

In the third cell, the elements appear that are present in both the first and the second cell.

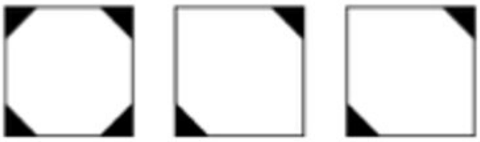

The top right and top left corners appear in both the first and second cell and are therefore mapped in the third cell. The other two corners from the first cell are only shown in this cell and are therefore not mapped in the third cell.
