# Supplementary material for: Test Preparation in Figural Matrices Tests: Focus on the Difficult Rules
Source: Front Psychol. 2021 Apr 15;12:619440. doi: 10.3389/fpsyg.2021.619440 (PMC8081851; doi:10.3389/fpsyg.2021.619440)
Supplement: Supplementary file 2 [file Table_1.docx]

Supplemental Material

*Table 1.* Rules used in the items

| Item | Addition | Subtraction | Single Element Addition | Intersection |
| --- | --- | --- | --- | --- |
| 1 | X | X |  |  |
| 2 | X |  | X |  |
| 3 | X |  |  | X |
| 4 |  | X | X |  |
| 5 |  | X |  | X |
| 6 |  |  | X | X |
| 7 | X | X | X |  |
| 8 | X | X | X |  |
| 9 | X | X | X |  |
| 10 | X | X | X |  |
| 11 | X | X |  | X |
| 12 | X | X |  | X |
| 13 | X | X |  | X |
| 14 | X | X |  | X |
| 15 | X |  | X | X |
| 16 | X |  | X | X |
| 17 | X |  | X | X |
| 18 | X |  | X | X |
| 19 |  | X | X | X |
| 20 |  | X | X | X |
| 21 |  | X | X | X |
| 22 |  | X | X | X |
| 23 | X | X | X | X |
| 24 | X | X | X | X |
| 25 | X | X | X | X |
| 26 | X | X | X | X |
