# Supplementary material for: Test Preparation in Figural Matrices Tests: Focus on the Difficult Rules
Source: Front Psychol. 2021 Apr 15;12:619440. doi: 10.3389/fpsyg.2021.619440 (PMC8081851; doi:10.3389/fpsyg.2021.619440)
Supplement: Supplementary file 3 [file Table_2.docx]

Supplemental Material

**Multigroup Confirmatory Factor Analyses**

**Configural Model**

lavaan 0.6-7 ended normally after 113 iterations

Estimator ML

Optimization method NLMINB

Number of free parameters 36

Number of observations per group:

easy training 68

no training 74

full training 67

difficult training 78

Model Test User Model:

Test statistic 0.000

Degrees of freedom 0

Test statistic for each group:

easy training 0.000

no training 0.000

full training 0.000

difficult training 0.000

Parameter Estimates:

Standard errors Standard

Information Expected

Information saturated (h1) model Structured

Group 1 [easy training]:

Latent Variables:

Estimate Std.Err z-value P(>|z|) Std.lv Std.all

overall =~

parcel1 1.000 0.315 0.936

parcel2 0.940 0.073 12.901 0.000 0.296 0.905

parcel3 1.045 0.072 14.476 0.000 0.329 0.943

Intercepts:

Estimate Std.Err z-value P(>|z|) Std.lv Std.all

.parcel1 0.627 0.041 15.378 0.000 0.627 1.865

.parcel2 0.562 0.040 14.165 0.000 0.562 1.718

.parcel3 0.599 0.042 14.172 0.000 0.599 1.719

overall 0.000 0.000 0.000

Variances:

Estimate Std.Err z-value P(>|z|) Std.lv Std.all

.parcel1 0.014 0.004 3.428 0.001 0.014 0.124

.parcel2 0.019 0.004 4.373 0.000 0.019 0.181

.parcel3 0.013 0.004 3.116 0.002 0.013 0.110

overall 0.099 0.020 5.071 0.000 1.000 1.000

Group 2 [no training]:

Latent Variables:

Estimate Std.Err z-value P(>|z|) Std.lv Std.all

overall =~

parcel1 1.000 0.347 0.960

parcel2 1.020 0.052 19.703 0.000 0.354 0.964

parcel3 0.943 0.059 15.848 0.000 0.327 0.917

Intercepts:

Estimate Std.Err z-value P(>|z|) Std.lv Std.all

.parcel1 0.533 0.042 12.695 0.000 0.533 1.476

.parcel2 0.533 0.043 12.505 0.000 0.533 1.454

.parcel3 0.549 0.041 13.250 0.000 0.549 1.540

overall 0.000 0.000 0.000

Variances:

Estimate Std.Err z-value P(>|z|) Std.lv Std.all

.parcel1 0.010 0.003 3.236 0.001 0.010 0.078

.parcel2 0.009 0.003 2.954 0.003 0.009 0.070

.parcel3 0.020 0.004 4.944 0.000 0.020 0.159

overall 0.120 0.022 5.580 0.000 1.000 1.000

Group 3 [full training]:

Latent Variables:

Estimate Std.Err z-value P(>|z|) Std.lv Std.all

overall =~

parcel1 1.000 0.302 0.978

parcel2 0.963 0.056 17.252 0.000 0.291 0.939

parcel3 0.986 0.069 14.187 0.000 0.298 0.895

Intercepts:

Estimate Std.Err z-value P(>|z|) Std.lv Std.all

.parcel1 0.682 0.038 18.062 0.000 0.682 2.207

.parcel2 0.663 0.038 17.509 0.000 0.663 2.139

.parcel3 0.694 0.041 17.076 0.000 0.694 2.086

overall 0.000 0.000 0.000

Variances:

Estimate Std.Err z-value P(>|z|) Std.lv Std.all

.parcel1 0.004 0.003 1.575 0.115 0.004 0.043

.parcel2 0.011 0.003 3.744 0.000 0.011 0.119

.parcel3 0.022 0.005 4.869 0.000 0.022 0.199

overall 0.091 0.017 5.480 0.000 1.000 1.000

Group 4 [difficult training]:

Latent Variables:

Estimate Std.Err z-value P(>|z|) Std.lv Std.all

overall =~

parcel1 1.000 0.229 0.930

parcel2 1.037 0.089 11.629 0.000 0.238 0.880

parcel3 0.961 0.083 11.591 0.000 0.220 0.879

Intercepts:

Estimate Std.Err z-value P(>|z|) Std.lv Std.all

.parcel1 0.798 0.028 28.596 0.000 0.798 3.238

.parcel2 0.759 0.031 24.829 0.000 0.759 2.811

.parcel3 0.817 0.028 28.809 0.000 0.817 3.262

overall 0.000 0.000 0.000

Variances:

Estimate Std.Err z-value P(>|z|) Std.lv Std.all

.parcel1 0.008 0.003 2.891 0.004 0.008 0.134

.parcel2 0.016 0.004 4.366 0.000 0.016 0.225

.parcel3 0.014 0.003 4.400 0.000 0.014 0.228

overall 0.053 0.010 5.281 0.000 1.000 1.000

**Weak Invariance Model**

lavaan 0.6-7 ended normally after 88 iterations

Estimator ML

Optimization method NLMINB

Number of free parameters 36

Number of equality constraints 6

Number of observations per group:

easy training 68

no training 74

full training 67

difficult training 78

Model Test User Model:

Test statistic 4.384

Degrees of freedom 6

P-value (Chi-square) 0.625

Test statistic for each group:

easy training 2.303

no training 1.270

full training 0.270

difficult training 0.541

Parameter Estimates:

Standard errors Standard

Information Expected

Information saturated (h1) model Structured

Group 1 [easy training]:

Latent Variables:

Estimate Std.Err z-value P(>|z|) Std.lv Std.all

overall =~

parcel1 1.000 0.317 0.939

parcel2 (.p2.) 0.990 0.031 31.482 0.000 0.314 0.917

parcel3 (.p3.) 0.980 0.035 28.406 0.000 0.311 0.928

Intercepts:

Estimate Std.Err z-value P(>|z|) Std.lv Std.all

.parcel1 0.627 0.041 15.320 0.000 0.627 1.858

.parcel2 0.562 0.042 13.536 0.000 0.562 1.642

.parcel3 0.599 0.041 14.754 0.000 0.599 1.789

overall 0.000 0.000 0.000

Variances:

Estimate Std.Err z-value P(>|z|) Std.lv Std.all

.parcel1 0.013 0.004 3.416 0.001 0.013 0.118

.parcel2 0.019 0.005 4.146 0.000 0.019 0.160

.parcel3 0.015 0.004 3.794 0.000 0.015 0.138

overall 0.101 0.018 5.437 0.000 1.000 1.000

Group 2 [no training]:

Latent Variables:

Estimate Std.Err z-value P(>|z|) Std.lv Std.all

overall =~

parcel1 1.000 0.348 0.962

parcel2 (.p2.) 0.990 0.031 31.482 0.000 0.345 0.959

parcel3 (.p3.) 0.980 0.035 28.406 0.000 0.341 0.924

Intercepts:

Estimate Std.Err z-value P(>|z|) Std.lv Std.all

.parcel1 0.533 0.042 12.669 0.000 0.533 1.473

.parcel2 0.533 0.042 12.765 0.000 0.533 1.484

.parcel3 0.549 0.043 12.787 0.000 0.549 1.486

overall 0.000 0.000 0.000

Variances:

Estimate Std.Err z-value P(>|z|) Std.lv Std.all

.parcel1 0.010 0.003 3.243 0.001 0.010 0.075

.parcel2 0.010 0.003 3.410 0.001 0.010 0.080

.parcel3 0.020 0.004 4.826 0.000 0.020 0.146

overall 0.121 0.021 5.776 0.000 1.000 1.000

Group 3 [full training]:

Latent Variables:

Estimate Std.Err z-value P(>|z|) Std.lv Std.all

overall =~

parcel1 1.000 0.300 0.976

parcel2 (.p2.) 0.990 0.031 31.482 0.000 0.297 0.943

parcel3 (.p3.) 0.980 0.035 28.406 0.000 0.294 0.893

Intercepts:

Estimate Std.Err z-value P(>|z|) Std.lv Std.all

.parcel1 0.682 0.038 18.141 0.000 0.682 2.216

.parcel2 0.663 0.038 17.230 0.000 0.663 2.105

.parcel3 0.694 0.040 17.245 0.000 0.694 2.107

overall 0.000 0.000 0.000

Variances:

Estimate Std.Err z-value P(>|z|) Std.lv Std.all

.parcel1 0.005 0.002 1.857 0.063 0.005 0.048

.parcel2 0.011 0.003 3.732 0.000 0.011 0.112

.parcel3 0.022 0.004 4.948 0.000 0.022 0.202

overall 0.090 0.016 5.558 0.000 1.000 1.000

Group 4 [difficult training]:

Latent Variables:

Estimate Std.Err z-value P(>|z|) Std.lv Std.all

overall =~

parcel1 1.000 0.231 0.933

parcel2 (.p2.) 0.990 0.031 31.482 0.000 0.228 0.867

parcel3 (.p3.) 0.980 0.035 28.406 0.000 0.226 0.886

Intercepts:

Estimate Std.Err z-value P(>|z|) Std.lv Std.all

.parcel1 0.798 0.028 28.501 0.000 0.798 3.227

.parcel2 0.759 0.030 25.491 0.000 0.759 2.886

.parcel3 0.817 0.029 28.286 0.000 0.817 3.203

overall 0.000 0.000 0.000

Variances:

Estimate Std.Err z-value P(>|z|) Std.lv Std.all

.parcel1 0.008 0.003 3.052 0.002 0.008 0.130

.parcel2 0.017 0.004 4.763 0.000 0.017 0.248

.parcel3 0.014 0.003 4.408 0.000 0.014 0.216

overall 0.053 0.009 5.713 0.000 1.000 1.000

**Strong Invariance Model**

lavaan 0.6-7 ended normally after 108 iterations

Estimator ML

Optimization method NLMINB

Number of free parameters 39

Number of equality constraints 15

Number of observations per group:

easy training 68

no training 74

full training 67

difficult training 78

Model Test User Model:

Test statistic 12.781

Degrees of freedom 12

P-value (Chi-square) 0.385

Test statistic for each group:

easy training 6.261

no training 3.961

full training 0.317

difficult training 2.243

Parameter Estimates:

Standard errors Standard

Information Expected

Information saturated (h1) model Structured

Group 1 [easy training]:

Latent Variables:

Estimate Std.Err z-value P(>|z|) Std.lv Std.all

overall =~

parcel1 1.000 0.317 0.933

parcel2 (.p2.) 0.982 0.030 32.780 0.000 0.311 0.915

parcel3 (.p3.) 0.992 0.033 30.276 0.000 0.314 0.931

Intercepts:

Estimate Std.Err z-value P(>|z|) Std.lv Std.all

.parcel1 (.p8.) 0.602 0.040 15.154 0.000 0.602 1.774

.parcel2 (.p9.) 0.579 0.039 14.768 0.000 0.579 1.703

.parcel3 (.10.) 0.611 0.040 15.459 0.000 0.611 1.813

overall 0.000 0.000 0.000

Variances:

Estimate Std.Err z-value P(>|z|) Std.lv Std.all

.parcel1 0.015 0.004 3.600 0.000 0.015 0.130

.parcel2 0.019 0.005 4.147 0.000 0.019 0.164

.parcel3 0.015 0.004 3.640 0.000 0.015 0.133

overall 0.100 0.018 5.430 0.000 1.000 1.000

Group 2 [no training]:

Latent Variables:

Estimate Std.Err z-value P(>|z|) Std.lv Std.all

overall =~

parcel1 1.000 0.348 0.962

parcel2 (.p2.) 0.982 0.030 32.780 0.000 0.342 0.956

parcel3 (.p3.) 0.992 0.033 30.276 0.000 0.345 0.926

Intercepts:

Estimate Std.Err z-value P(>|z|) Std.lv Std.all

.parcel1 (.p8.) 0.602 0.040 15.154 0.000 0.602 1.665

.parcel2 (.p9.) 0.579 0.039 14.768 0.000 0.579 1.620

.parcel3 (.10.) 0.611 0.040 15.459 0.000 0.611 1.641

overall -0.060 0.057 -1.047 0.295 -0.172 -0.172

Variances:

Estimate Std.Err z-value P(>|z|) Std.lv Std.all

.parcel1 0.010 0.003 3.229 0.001 0.010 0.075

.parcel2 0.011 0.003 3.557 0.000 0.011 0.086

.parcel3 0.020 0.004 4.755 0.000 0.020 0.143

overall 0.121 0.021 5.784 0.000 1.000 1.000

Group 3 [full training]:

Latent Variables:

Estimate Std.Err z-value P(>|z|) Std.lv Std.all

overall =~

parcel1 1.000 0.300 0.976

parcel2 (.p2.) 0.982 0.030 32.780 0.000 0.295 0.941

parcel3 (.p3.) 0.992 0.033 30.276 0.000 0.298 0.896

Intercepts:

Estimate Std.Err z-value P(>|z|) Std.lv Std.all

.parcel1 (.p8.) 0.602 0.040 15.154 0.000 0.602 1.958

.parcel2 (.p9.) 0.579 0.039 14.768 0.000 0.579 1.848

.parcel3 (.10.) 0.611 0.040 15.459 0.000 0.611 1.839

overall 0.081 0.054 1.499 0.134 0.271 0.271

Variances:

Estimate Std.Err z-value P(>|z|) Std.lv Std.all

.parcel1 0.004 0.002 1.860 0.063 0.004 0.047

.parcel2 0.011 0.003 3.795 0.000 0.011 0.114

.parcel3 0.022 0.004 4.919 0.000 0.022 0.198

overall 0.090 0.016 5.563 0.000 1.000 1.000

Group 4 [difficult training]:

Latent Variables:

Estimate Std.Err z-value P(>|z|) Std.lv Std.all

overall =~

parcel1 1.000 0.230 0.933

parcel2 (.p2.) 0.982 0.030 32.780 0.000 0.226 0.863

parcel3 (.p3.) 0.992 0.033 30.276 0.000 0.228 0.887

Intercepts:

Estimate Std.Err z-value P(>|z|) Std.lv Std.all

.parcel1 (.p8.) 0.602 0.040 15.154 0.000 0.602 2.442

.parcel2 (.p9.) 0.579 0.039 14.768 0.000 0.579 2.210

.parcel3 (.10.) 0.611 0.040 15.459 0.000 0.611 2.375

overall 0.196 0.048 4.096 0.000 0.853 0.853

Variances:

Estimate Std.Err z-value P(>|z|) Std.lv Std.all

.parcel1 0.008 0.003 3.030 0.002 0.008 0.130

.parcel2 0.018 0.004 4.823 0.000 0.018 0.256

.parcel3 0.014 0.003 4.373 0.000 0.014 0.214

overall 0.053 0.009 5.719 0.000 1.000 1.000

**Strict Invariance Model**

lavaan 0.6-7 ended normally after 40 iterations

Estimator ML

Optimization method NLMINB

Number of free parameters 39

Number of equality constraints 24

Number of observations per group:

easy training 68

no training 74

full training 67

difficult training 78

Model Test User Model:

Test statistic 27.544

Degrees of freedom 21

P-value (Chi-square) 0.154

Test statistic for each group:

easy training 10.177

no training 6.466

full training 6.984

difficult training 3.916

Parameter Estimates:

Standard errors Standard

Information Expected

Information saturated (h1) model Structured

Group 1 [easy training]:

Latent Variables:

Estimate Std.Err z-value P(>|z|) Std.lv Std.all

overall =~

parcel1 1.000 0.317 0.957

parcel2 (.p2.) 0.980 0.031 31.383 0.000 0.311 0.931

parcel3 (.p3.) 0.991 0.033 30.272 0.000 0.315 0.922

Intercepts:

Estimate Std.Err z-value P(>|z|) Std.lv Std.all

.parcel1 (.p8.) 0.609 0.040 15.400 0.000 0.609 1.836

.parcel2 (.p9.) 0.580 0.039 14.849 0.000 0.580 1.735

.parcel3 (.10.) 0.615 0.040 15.537 0.000 0.615 1.801

overall 0.000 0.000 0.000

Variances:

Estimate Std.Err z-value P(>|z|) Std.lv Std.all

.parcel1 (.p4.) 0.009 0.002 5.798 0.000 0.009 0.084

.parcel2 (.p5.) 0.015 0.002 8.097 0.000 0.015 0.133

.parcel3 (.p6.) 0.018 0.002 8.695 0.000 0.018 0.151

overall 0.101 0.018 5.513 0.000 1.000 1.000

Group 2 [no training]:

Latent Variables:

Estimate Std.Err z-value P(>|z|) Std.lv Std.all

overall =~

parcel1 1.000 0.346 0.963

parcel2 (.p2.) 0.980 0.031 31.383 0.000 0.340 0.941

parcel3 (.p3.) 0.991 0.033 30.272 0.000 0.343 0.933

Intercepts:

Estimate Std.Err z-value P(>|z|) Std.lv Std.all

.parcel1 (.p8.) 0.609 0.040 15.400 0.000 0.609 1.693

.parcel2 (.p9.) 0.580 0.039 14.849 0.000 0.580 1.606

.parcel3 (.10.) 0.615 0.040 15.537 0.000 0.615 1.670

overall -0.065 0.057 -1.151 0.250 -0.189 -0.189

Variances:

Estimate Std.Err z-value P(>|z|) Std.lv Std.all

.parcel1 (.p4.) 0.009 0.002 5.798 0.000 0.009 0.072

.parcel2 (.p5.) 0.015 0.002 8.097 0.000 0.015 0.114

.parcel3 (.p6.) 0.018 0.002 8.695 0.000 0.018 0.129

overall 0.120 0.021 5.783 0.000 1.000 1.000

Group 3 [full training]:

Latent Variables:

Estimate Std.Err z-value P(>|z|) Std.lv Std.all

overall =~

parcel1 1.000 0.298 0.952

parcel2 (.p2.) 0.980 0.031 31.383 0.000 0.292 0.923

parcel3 (.p3.) 0.991 0.033 30.272 0.000 0.296 0.913

Intercepts:

Estimate Std.Err z-value P(>|z|) Std.lv Std.all

.parcel1 (.p8.) 0.609 0.040 15.400 0.000 0.609 1.942

.parcel2 (.p9.) 0.580 0.039 14.849 0.000 0.580 1.829

.parcel3 (.10.) 0.615 0.040 15.537 0.000 0.615 1.897

overall 0.078 0.054 1.443 0.149 0.262 0.262

Variances:

Estimate Std.Err z-value P(>|z|) Std.lv Std.all

.parcel1 (.p4.) 0.009 0.002 5.798 0.000 0.009 0.095

.parcel2 (.p5.) 0.015 0.002 8.097 0.000 0.015 0.148

.parcel3 (.p6.) 0.018 0.002 8.695 0.000 0.018 0.167

overall 0.089 0.016 5.444 0.000 1.000 1.000

Group 4 [difficult training]:

Latent Variables:

Estimate Std.Err z-value P(>|z|) Std.lv Std.all

overall =~

parcel1 1.000 0.231 0.923

parcel2 (.p2.) 0.980 0.031 31.383 0.000 0.226 0.880

parcel3 (.p3.) 0.991 0.033 30.272 0.000 0.229 0.865

Intercepts:

Estimate Std.Err z-value P(>|z|) Std.lv Std.all

.parcel1 (.p8.) 0.609 0.040 15.400 0.000 0.609 2.436

.parcel2 (.p9.) 0.580 0.039 14.849 0.000 0.580 2.256

.parcel3 (.10.) 0.615 0.040 15.537 0.000 0.615 2.327

overall 0.191 0.048 3.996 0.000 0.829 0.829

Variances:

Estimate Std.Err z-value P(>|z|) Std.lv Std.all

.parcel1 (.p4.) 0.009 0.002 5.798 0.000 0.009 0.149

.parcel2 (.p5.) 0.015 0.002 8.097 0.000 0.015 0.226

.parcel3 (.p6.) 0.018 0.002 8.695 0.000 0.018 0.251

overall 0.053 0.009 5.682 0.000 1.000 1.000

**Model Comparison**

Chi-Squared Difference Test

Df AIC BIC Chisq Chisq diff Df diff Pr(>Chisq)

Config 0 -353.64 -221.90 0.000

Weak 6 -361.25 -251.47 4.384 4.3840 6 0.62486

Strong 12 -364.86 -277.03 12.781 8.3975 6 0.21040

Strict 21 -368.09 -313.20 27.544 14.7624 9 0.09767 .

---

Signif. codes: 0 ‘***’ 0.001 ‘**’ 0.01 ‘*’ 0.05 ‘.’ 0.1 ‘ ’ 1
